# Modulation of allergic airways disease employing bio-mimetic nanoparticles with TLR agonists

**DOI:** 10.3389/falgy.2025.1633293

**Published:** 2025-08-29

**Authors:** Melanie Cristine Scalise, Seyran Mutlu, Céline Ferrié, Mario Amacker, Christophe von Garnier, Philip Stumbles, Fabian Blank

**Affiliations:** ^1^ Lung Precision Medicine (LPM), Department for BioMedical Research (DBMR), University of Bern, Bern, Switzerland; ^2^ Department for Pulmonary Medicine, Allergology and Clinical Immunology, Inselspital, Bern University Hospital, University of Bern, Bern, Switzerland; ^3^ Graduate School for Cellular and Biomedical Sciences, University of Bern, Bern, Switzerland; ^4^ Division of Pulmonology, Department of Medicine, Centre Hospitalier Universitaire Vaudois (CHUV) and University of Lausanne, Lausanne, Switzerland; ^5^ College of Science, Health, Engineering and Education, Murdoch University, Perth, WA, Australia; ^6^ Telethon Kids Institute, Perth Children's Hospital, Perth, WA, Australia

**Keywords:** allergic airways model, asthma, dendritic cells, bio-mimetic nanoparticles, TLR7/8 agonists

## Abstract

**Introduction:**

Allergic asthma is characterized by airway hyperresponsiveness due to a biased Th2 immune response against harmless environmental substances. While most current treatments alleviate symptoms without altering the disease's progression, allergen-specific immunotherapy (AIT) is the only clinically approved strategy known to modify the natural course of allergic disease. However, AIT has limitations, highlighting the need for improved formulations that provide safer, faster, and more effective immune modulation.

**Methods:**

In this study, we designed bio-mimetic nanoparticles and evaluated their effects in a mouse model of experimental allergic inflammatory airways disease (EAIAD). Mice were sensitized with ovalbumin (OVA) and treated with liposomes or virosomes conjugated with OVA and the TLR7/8 agonist 3M-052. Lung function, inflammatory cell recruitment, cytokine profiles, and immunoglobulin levels were analyzed post-treatment.

**Results:**

Among the tested formulations, liposomes co-delivering OVA and 3M-052 (Lipo-OVA) led to partial improvements in lung mechanics, including lower airway resistance (Rrs) and preserved forced expiratory volume (FEV0.1). Immune profiling revealed formulation-specific effects on eosinophil and macrophage populations, and modest shifts in cytokine secretion patterns. However, no formulation fully resolved airway inflammation or significantly reduced Th2 cytokines or total IgE levels.

**Discussion:**

These findings support the feasibility of nanoparticle-based AIT strategies, while also highlighting the need for further optimization to enhance efficacy, minimize sensitization, and promote sustained long-term immune tolerance.

## Introduction

Asthma is a globally significant respiratory disease affecting more than 300 million people with its prevalence expected to increase (1, 2). The most common phenotype is allergic asthma, which accounts for more than half of all asthma cases (3, 4). Allergic asthma is triggered by inhaled allergens such as pollen, house dust mites, pet dander or mold (5) and is characterized by reversible airway obstruction, eosinophilic inflammation, mucus hypersecretion and airway hyperresponsiveness (6, 7). These hallmark features are induced by a maladaptive Th2 immune response (8), orchestrated by pulmonary dendritic cells (DC). DC capture inhaled allergens and drive the downstream immune response by inducing effector T cells and IgE-producing B cells in the lung-draining lymph nodes (LDLN) (9–12).

During sensitization, allergens are inhaled and deposited on the airway epithelium, activating epithelial cells either through their proteolytic activity (4, 13) or through pattern-recognition receptors such as Toll-like receptor 4 (TLR4) (6, 14). Current treatments for allergic asthma primarily involve with life-long inhalation of corticosteroids to control inflammation, often in combination with long-acting beta agonists (LABA) as bronchodilators, or allergen immunotherapy (15). However, while most treatments primarily aim to relieve symptoms, allergen-specific immunotherapy (AIT) is recognized as the only disease-modifying approach capable of altering the course of allergic diseases and providing long-term benefit. Nevertheless, AIT has limitations, including prolonged treatment regimens, extract standardization issues, and safety concerns, that motivate the development of more advanced and targeted strategies (16).

Over the past two decades, allergen immunotherapy has emerged as a widely accepted approach for treating allergic rhinitis and asthma. This method aims to modulate and/or desensitize the allergic immune response via subcutaneous or sublingual route, targeting both humoral and cellular immunity. Though, this is a well characterized and promising approach, allergen immunotherapy has several limitations, including prolonged treatment durations, a lack of standardized allergen extracts, and safety concerns. To overcome these challenges, further research is needed to develop potent antigen carriers, purified synthetic or recombinant allergens, and adjuvants capable of accelerating immunomodulation for more effective and safer therapies.

Clinically approved bio-mimetic nanocarriers, such as liposomes or virosomes derived from empty influenza virus envelopes, are considered safe for human vaccine applications due to their lack of genetic material. In this study, we aimed to explore the immunomodulatory potential of bio-mimetic nanoparticles in an optimized EAIAD mouse model. By combining allergen (OVA) with the TLR7/8 agonists 3M-052 on the nanoparticle surface, we sought to reprogram the allergic immune response by shifting the Th2-biased inflammation, thereby restoring Th1/Th2 homeostasis (17–19). We hypothesize that this shift in immune response would reduce allergic asthma symptoms (20, 21). To investigate this novel therapeutic approach, we employed bio-mimetic nanocarriers, specifically liposomes and reconstituted influenza virosomes (henceforth referred to as virosomes), with allergen (ovalbumin, OVA) and adjuvant (TLR7/8 agonist 3M-052) anchored to their lipid bilayer. These nanocarriers were systematically evaluated for their effects on lung function, airway inflammation, and immune polarization. As promising candidates for therapeutic vaccination, they can be engineered to target pulmonary DC, mimic pathogen characteristics, and efficiently display a combination of allergen and adjuvant. Previous studies from our laboratory have demonstrated that virosomes can induce robust antigen-specific T cell responses, supporting their potential as an effective therapeutic strategy (10, 22).

In this study, we explored the potential of clinically relevant liposomes and virosomes as delivery platforms for allergen-specific immunotherapy, co-presenting the model antigen OVA and the TLR7/8 agonist 3M-052. Using an optimized murine EAIAD model without aluminum adjuvants, we assessed lung function, immune cell infiltration, cytokine profiles, and allergen-reactive antibodies following treatment. This design allowed for a comparative evaluation of formulation-dependent immunomodulatory effects and their relevance to allergic airway inflammation. This bio-mimetic nanoparticle-based strategy builds on AIT principles (23, 24) by enhancing antigen delivery and immune modulation, offering insights into how formulation influences therapeutic potential and informing future efforts to optimize safety, efficacy, and duration of immunotherapy.

## Materials and methods

### Ethical approval

All animal experiments were approved by the Swiss Federal Veterinary Office and the Cantonal Ethical Committee for Animal Experiments (Amt für Landwirtschaft und Natur des Kantons Bern) under permission number BE 71/18. Mice were housed and handled in accordance with the “Principles of Laboratory Animal Care” established by the National Society for Medical Research and the “Guide for the Care and Use of Laboratory Animals” issued by the Institute of Laboratory Animal Research. All procedures complied with the ethical guidelines of the European Convention for the Protection of Vertebrate Animals Used for Experimental and Other Scientific Purposes.

### Mice

Female BALB/c mice obtained from JANVIER LABS (Janvier labs Le Genest Saint Isle Saint Berthevin, France) were used at 8–12 weeks of age and housed under clean conditions and on non-allergic, OVA-free food (Granovit AG) at the Department of Biomedical Research (University of Bern).

### EAIAD mouse model

**Figure 1:**
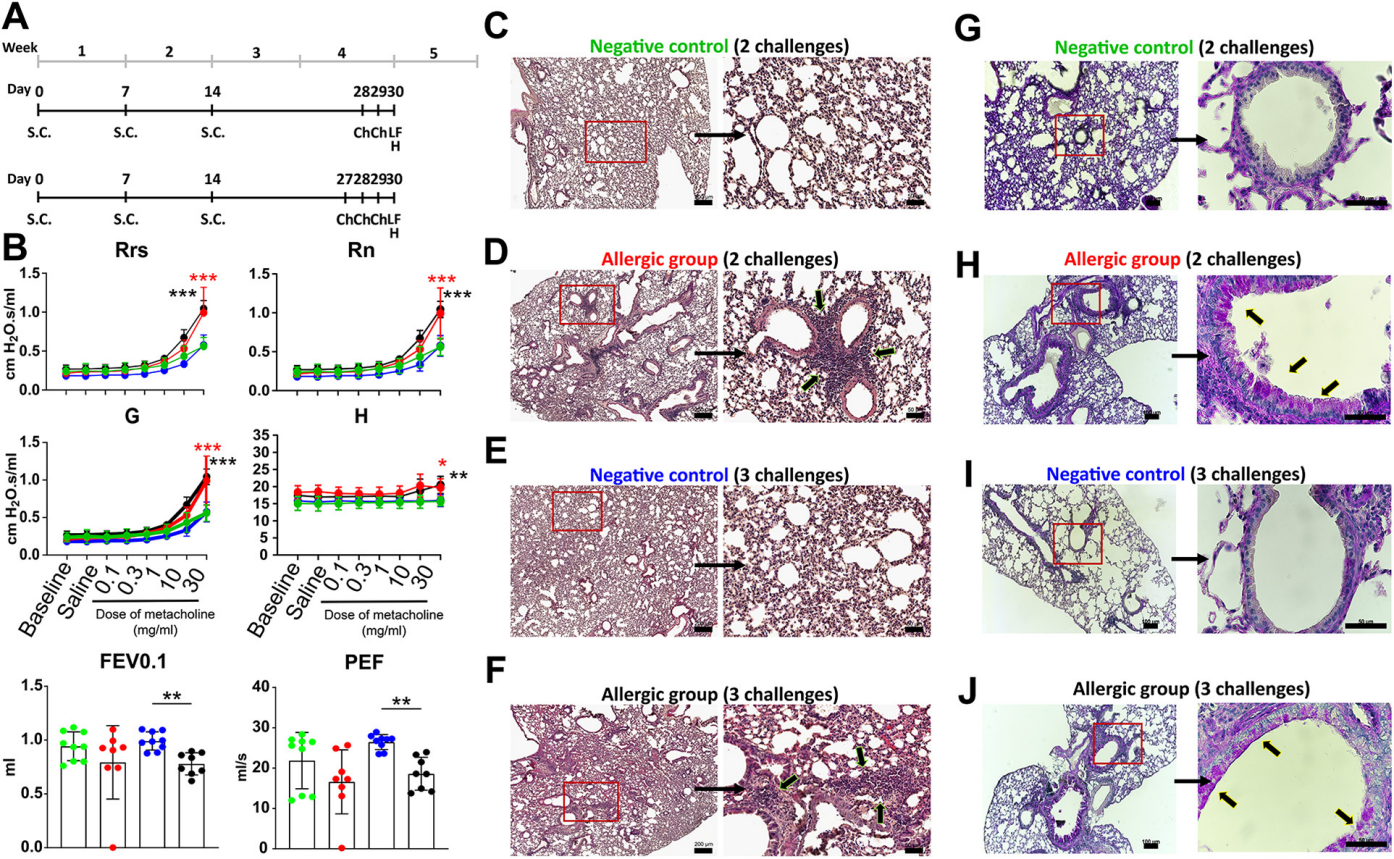
Experimental design and lung function analysis of the EAIAD mouse model. **(A)** Timeline of the EAIAD protocol. Mice were sensitized subcutaneously with ovalbumin (OVA) or saline (control) on days 0, 7, and 14. Animals were then challenged with aerosolized 1% OVA/NaCl either twice (on days 28 and 29) or three times (on days 27, 28, and 29), followed by endpoint analyses 24 h after the final challenge, including organ and blood collection and lung function assessment. **(B)** Lung function parameters were measured using the FlexiVent FX system (SCIREQ): Respiratory system resistance (Rrs), airway resistance (Rn), tissue damping **(G)**, tissue elastance **(H)**, forced expiratory volume in 0.1 s (FEV0.1), and peak expiratory flow (PEF). Sensitized mice with two or three OVA challenges [Allergic group (2), Allergic group (3)] are compared to corresponding negative controls [Negative group (2), Negative group (3)]. Data are presented as mean ± SEM; *n* = 3 animals per group, each shown with three technical replicates. *p < 0.05, **p < 0.01, ***p < 0.001 indicate significance. **(C–F)** Hematoxylin and eosin (H&E) staining of paraffin-embedded lung tissue sections: **(C)** negative control, two times challenge; **(D)** sensitized, two times challenge; **(E)** negative control, three times challenge; **(F)** sensitized, three times challenge. Left panels: overview images 5x magnification (scale bar = 200 µm). Right panels: magnified insets 20x magnification (scale bar = 50 µm), showing regions of increased inflammatory cell infiltration (arrows). Periodic acid- Schiff (PAS) staining in paraffin-embedded lung sections from: **(G)** negative control, two times challenge, **(H)** sensitized, two times challenge, **(I)** negative control, three times challenge, **(J)** sensitized, three times challenge. Left panels: overview images 10x magnification (scale bar = 100 µm). Right panels: magnified insets 60x magnification (scale bar = 50 µm), showing regions of mucus producing cells (arrows).

As shown in Figure 1A, mice were sensitized by subcutaneous (s.c.) injection with 200 µl OVA solution (0.05 mg/ml, Hyglos) on days 0, 7 and 14. No adjuvant (e.g., aluminum hydroxide) was used in order to establish an adjuvant-free model of allergic airway inflammation.

Mice were then challenged either two or three times with OVA/NaCl (1%) aerosol, delivered with an ultrasonic nebulizer (SonoDrop; MPV Trauma) for 30 min on consecutive days starting on day 27. An additional group which received s.c. PBS and was challenged with OVA/NaCl aerosol, served as control group. Lung function measurements and organ harvest was performed twenty-four hours after the last challenge.

### OVA-PE conjugation and virosome and liposome formulation

The model antigen OVA (5 mg/ml in PBS, Hyglos) was thiolated using 2-iminothiolane (Traut's reagent; Merck KGaA) and subsequently conjugated to 1,2-dipalmitoyl-sn-glycero-3-phosphoethanolamine-N-[4-(p-maleinimidomethyl)cyclohexane-carboxamide] (N-MCC-DPPE; Corden Pharma) to generate a lipid-anchored OVA construct (OVA-DPPE), as previously described (25). This conjugation strategy enabled stable incorporation of OVA on the outer membrane of virosomes or liposomes. Virosomes were formulated from an inactivated Influenza virus H1N1 strain A/Brisbane/59/2007 (CSL Seqirus; provided by Mymetics SA). Liposomes were generated using an identical process, omitting viral membrane components. The TLR7/8 agonist 3M-052 used as adjuvant (1.5 mg/ml in DMSO; 3M Company; provided by Mymetics SA) was added during the particle formulation process to the virosomal and liposomal excipient mix together with the OVA-DPPE conjugate, as previously described (25). All formulations were characterized prior to use as previously described (25, 26). The final formulated virosomes contained approximately nine times more OVA than hemagglutinin (HA). Because the OVA antigen was covalently attached to the nanoparticle surface, we report integration efficiency (IE%), calculated by quantifying unbound OVA in the supernatant via dot blot, interpolated against a standard curve. This reflects the proportion of input OVA successfully incorporated onto the particle surface. For virosomal formulations, hemagglutinin (HA) content, reflecting viral membrane protein integration, was semi-quantified via dot blot and included in Supplementary Table S1. On average, the final virosomal formulations contained approximately nine times more OVA than HA.

### Analysis of virosomes and liposomes employing cryo-transmission electron microscopy (cryo-TEM)

For cryo-TEM, 4 µl of each sample with a particle concentration of 1 × 10^9^–1 × 10^10^/ml was applied to a glow discharged grid (30 min 10 mA; Baltzers CTA 010). Vitrification was performed using a FEI Vitrobot Mark IV. Sample was applied to the grids at 4°C for 10 min at 100% humidity followed by 4 min blotting. Vitrified grids were stored in liquid nitrogen prior to acquisition. Imaging was performed on a FEI Tecnai F20 equipped with a Falcon III camera with a total dose not exceeding 60 e−/Å2. In addition, particle size was measured by dynamic light scattering (DLS) using a Zetasizer Nano S (Malvern Instruments) at 25°C in PBS. The Z-average diameter and polydispersity index (PDI) were automatically calculated using the instrument's software via the cumulants analysis method, as defined by ISO 13321. Results are reported in Supplementary Table S1.

### Treatment approach employing bio-mimetic nanoparticles

**Figure 2:**
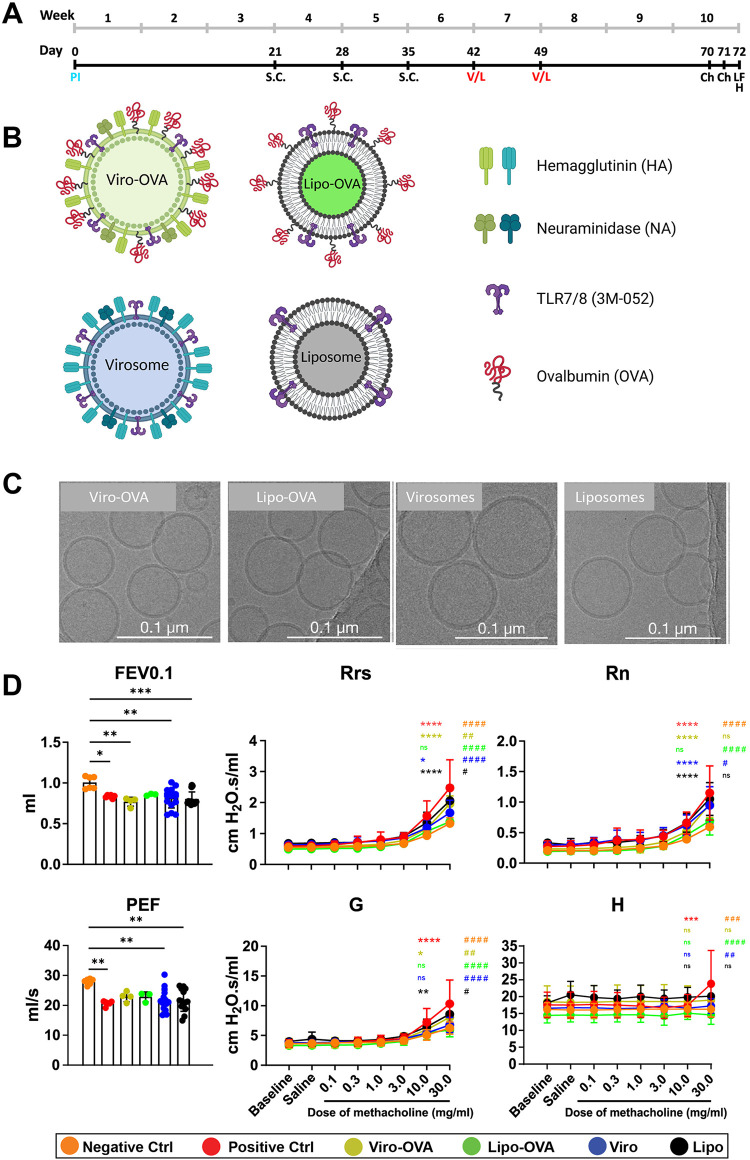
Bio-mimetic nanoparticle therapy in a mouse model of EAIAD. **(A)** Experimental timeline of the EAIAD model and therapeutic intervention. Mice were pre-immunized on day 0 and sensitized subcutaneously (s.c.) with OVA or saline (control) on days 21, 28, and 35. Nanoparticle treatments were administered on days 42 and 49. Mice were challenged with aerosolized OVA on days 70 and 71, followed by lung function measurement and tissue/blood collection on day 72. **(B)** Schematic representation of nanoparticle formulations: (i) Viro-OVA, virosomes with influenza HA/NA proteins and surface-conjugated OVA; (ii) Lipo-OVA, liposomes conjugated with OVA, no viral proteins; (iii) Virosomes, with HA/NA proteins without OVA; and (iv) Liposomes, plain lipid vesicles without HA/NA or OVA. Created with BioRender.com. **(C)** Representative cryo-EM images of each nanoparticle formulation. Scale bars: 0.1 μm. **(D)** Lung function parameters: forced expiratory volume in 0.1s (FEV0.1), peak expiratory flow (PEF), total respiratory system resistance (Rrs), airway resistance (Rn), tissue damping **(G)**, and tissue elastance **(H)** measured using FlexiVent FX system. Data are shown as mean±SEM. Asterisks indicate significance vs. negative control (*p < 0.05, **p < 0.01, ***p < 0.001). Hashtags indicate significance vs. positive control (^#^*p* < 0.05, ^##^*p* < 0.01, ^###^*p* < 0.001).

As shown in Figure 2A, mice were pre-immunized by s.c. injection with 100 µl inactivated Influenza A/Brisbane/59/2007 H1N1 virus (0.01 mg/ml HA, provided by Mymetics SA) on day 0 (27, 28). This step was included to mimic pre-existing anti-influenza immunity, which is common in the human population due to prior infection or vaccination. The presence of such immunity is relevant for virosome-based formulations that present influenza membrane proteins and may enhance nanoparticle uptake through improved immune recognition.

Mice were then sensitised with OVA, as described above on days 21, 28 and 35. Treatments were applied by s.c. injection with 100 µl virosome alone, liposome alone, OVA conjugated to virosome or OVA coupled to liposome solutions ([OVA] = 30 µg/ml; [HA] = 3.36 µg/ml) on days 46 and 53. Finally, mice were challenged two times as described above. Lung function measurements and organ harvest were performed 24 h after the last challenge.

### Single cell preparation

Preparation of single cell suspensions for flow cytometry followed the previously described protocol (11). In brief, organs were collected and placed in ice-cold GKN + 10% FBS, then finely minced with a scalpel blade. The minced organs underwent incubation in a mixture of Collagenase IV (Worthington, USA) and DNAse I (Sigma Aldrich, USA) in GKN + 10% FBS for 90 min for lung parenchyma and trachea, and 30 min for LDLN, utilizing a shaking water bath at 37°C. The digested organs were filtered through a 70 µm mesh, centrifuged at 1,640 rpm for 8 min, and the resulting pellets were resuspended in ice-cold GKN + 5% FBS. Subsequently, samples were maintained on ice until further processing.

### Flow cytometry

Preparation and labelling of single cell suspensions was done as previously described (10). To reduce inter-animal variability and ensure sufficient cell yield, tissue samples from two mice were pooled per data point prior to staining and analysis. Isolated cells were incubated on ice for 10 min with FcR block (anti-mouse; Miltenyi Biotec), to block unspecific FC receptor binding. Viability staining was performed by using Zombie Violet Fixable Viability (BioLegend) for 30 min on ice. All Antibodies (Abs) were obtained from BioLegend, unless indicated otherwise with appropriate isotype control: CD45-Brilliant Violet 650, CD11c-Brilliant Violet 605, I-A/I-E (MHC class II)-Pacific Blue, CD11b-APC eFluor 780 (eBioscience), F4/80-PE (eBioscience) and CD103-Brilliant Ultraviolet 395 (BD Pharmingen), Siglec-F-PE-CF594 (BD Pharmingen), Ly6G-Brilliant Violet 711, CD40-PE-Cy 7 (Supplementary Figure S1). Surface marker-labelled cells were fixed for 10 min at room temperature with 1% formalin solution. Acquisition was performed by using a SORP LSR II (BD Bioscience) flow cytometer and data was analysed with FlowJo software (10.7.1).

### Lung function measurements

Measurements were performed as previously described (12, 29). Lung resistance was analysed by using the FlexiVent FX system (ScireQ). Mice were anesthetized by intraperitoneal injection of 200 mg/kg ketamine and 10 mg/kg xylazine. After eight min, mice were tracheotomized and mechanically ventilated at a rate of 150 breaths per min (normal ventilation) or 250 breaths per minute (hyperventilation) and a tidal volume of 10 ml per kg body weight. Airway hyperresponsiveness was measured by administrating increasing doses of methacholine, ranging from 0.1–30 mg/ml (Sigma).

### Measurement of total IgE

Blood was collected via cardiac puncture, and serum was prepared for immunoglobulin analysis. Total IgE was measured using a custom sandwich ELISA. Briefly, 96-well NUNC Maxisorp plates were coated overnight at 4°C with 2 µg/ml rat anti-mouse IgE (BD Pharmingen). After washing with 0.1% Tween-20 in PBS and blocking with 1% BSA in PBS, diluted mouse serum or monoclonal mouse anti-OVA IgE (Bio-Rad, MCA2259) was added and incubated for 1 h at room temperature. Bound total IgE was detected using 1 µg/ml HRP-conjugated goat anti-mouse IgE (Bio-Rad, STAR110P), followed by colorimetric development with o-phenylenediamine (OPD; Sigma) and 0.04% hydrogen peroxide. The reaction was stopped with 50 µl of 2N sulfuric acid, and absorbance was measured at 492 nm using a Tecan Infinite M1000 plate reader.

### Measurement of total immunoglobulin isotypes

Total immunoglobulin isotypes were quantified using the ProcartaPlex Mouse Antibody Isotyping Panel 7-Plex (Thermo Fisher), according to the manufacturer's instructions. Data were acquired using a Bio-Plex 3D suspension array system (Luminex xMAP technology; Bio-Rad).

### Cytokine concentrations

The broncho alveolar lavage fluid (BALF) was concentrated using a Pierce™ Protein Concentrator PES 3K MWCO (Thermo Fisher) according to manufacturer's manual instructions. Quantification of IL-1β, IL-4, IL-5, IL-12p70, IL-13, TNF-α and IL-10 concentrations in the supernatant of BAL was performed using a Th1/Th2/Th9/Th17/Th22/Treg Cytokine 17-Plex Mouse ProcartaPlex Panel assay kit (Invitrogen, Thermo Fisher). The assay was measured by a Bio-Plex 3D suspension array system employing Luminex xMap technology (Bio-Rad).

### Histology

Left lobes of lungs were fixed in 10% formalin and incubated overnight at 4°C, followed by a dehydration process of 50% ETOH for 30 min at 4°C. Samples were then stored in 70% ETOH at 4°C until embedding into paraffin. Prepared sections (5 µm) were stained with H&E and PAS reagents according to standardized protocols and analysed with a slide scanner (3DHistech Panoramic 250 Flash II).

### Statistics

Depending on data distributions an ordinary one-way ANOVA or Kruskal–Wallis test was performed to calculate significance between the different control and treatment groups. *P* < 0.05 was considered as significant. GraphPad Prism (version 9.2.0) was used for statistical analysis of data and graph generation.

## Results

### The number of challenges affects the severity of EAIAD in lung function and tissue structure

To assess how the number of OVA aerosol challenges influences the severity of EAIAD, we evaluated lung function and lung tissue morphology. Mice were sensitized subcutaneously with OVA or saline (control) on days 0, 7, and 14 and subsequently challenged either two or three times with aerosolized 1% OVA/NaCl starting on day 27. (Figure 1A). Lung function was analyzed using the FlexiVent FX system (SCIREQ). Respiratory system resistance (Rrs) was significantly increased in both OVA-sensitized groups (two and three challenges) compared to their respective controls following increasing doses of methacholine. Similarly, central airway resistance (Rn), reflecting upper airway resistance, was significantly elevated in both sensitized groups relative to controls. Tissue damping (G), a measure of resistance at the alveolar level, also showed a significant increase in both OVA groups. For tissue elastance (H), no significant differences were observed across most methacholine concentrations; however, a significant increase was detected in the group challenged three times at the highest methacholine dose (30 mg/ml). Forced expiratory volume in 0.1 s (FEV0.1), an analog to human FEV1 (30), was significantly reduced in the three times challenged allergic group compared to its control. Similarly, peak expiratory flow (PEF) was significantly lower in this group (Figure 1B).

To evaluate inflammatory cell infiltration, lung tissue sections were stained with hematoxylin and eosin (H&E). Both OVA-sensitized groups challenged two- or three-times displayed areas of dense cellular infiltration not present in their respective control groups (Figures 1C–F), confirming the presence of allergen-induced lung inflammation. Periodic acid–Schiff (PAS) staining revealed the presence of PAS-positive goblet cells along the airway epithelium in mice that underwent two or three OVA aerosol challenges. In these groups, intra-luminal PAS-positive material was also observed, consistent with visible mucus accumulation. In contrast, airway sections from control animals displayed minimal or no PAS-positive staining in the epithelium or lumen (Figures 1G–J).

### Induction of cellular allergic response depending on number of challenges

**Figure 3:**
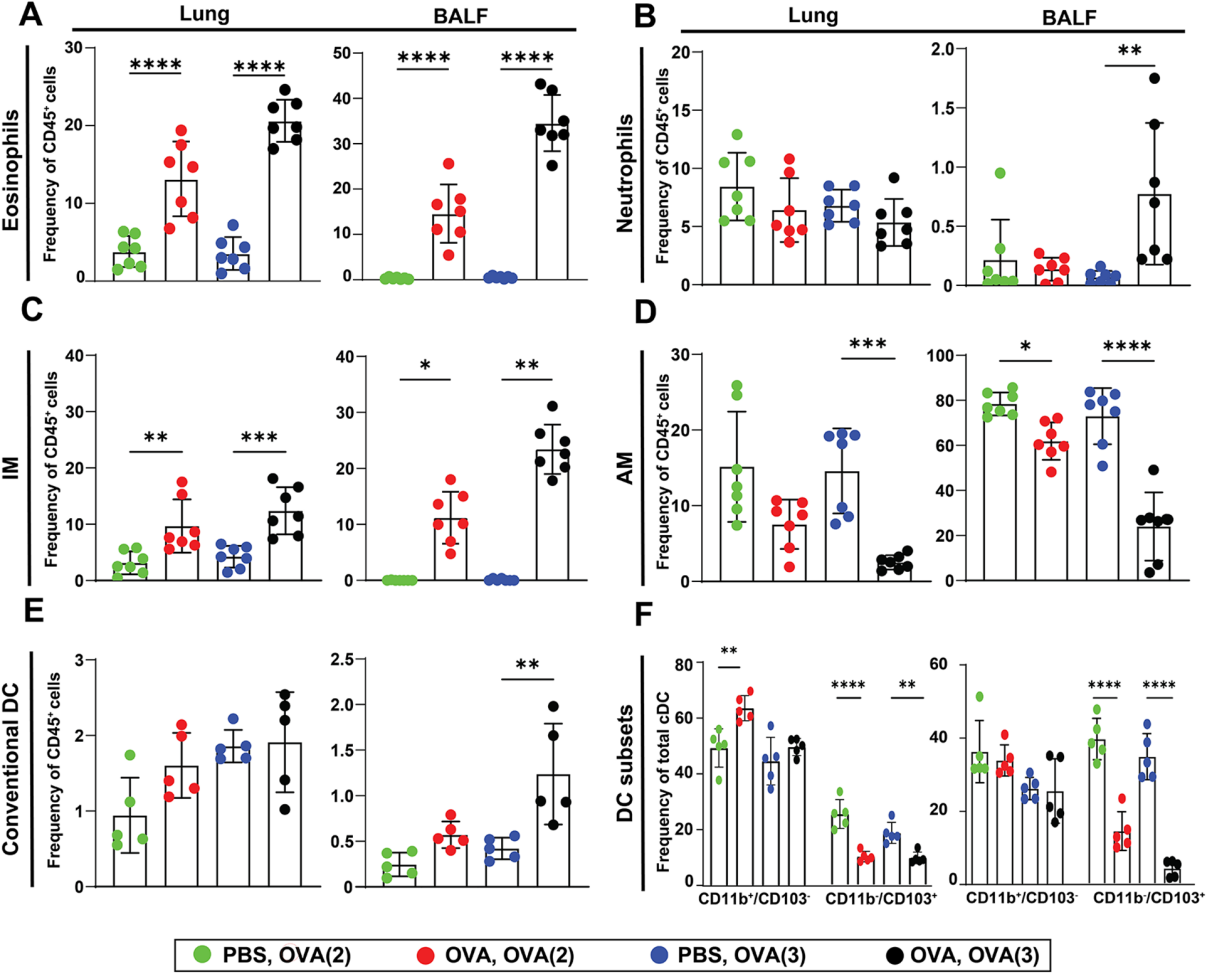
Immune cell profiles in the lungs and BALF after OVA challenges. Flow cytometric analysis of immune cell frequencies (within CD45^+^ cells) in sensitized and control mice after two or three OVA aerosol challenges (see Figure 1A). Frequencies of immune cell subsets within total CD45^+^ cells are shown for: **(A)** eosinophils, **(B)** neutrophils, **(C)** interstitial macrophages (IM), **(D)** alveolar macrophages (AM), **(E)** total conventional dendritic cells (cDCs), and **(F)** dendritic cell subsets (CD11b^+^/CD103^−^, and CD11b^−^/CD103^+^). Each data point represents a pooled sample from two animals, resulting in *n* = 7 pooled samples per group, except in panels E and F, where *n* = 5 pooled samples due to technical limitations. Data shows mean ± SEM. *p < 0.05, **p < 0.01, ***p < 0.001 and ****p < 0.0001 show significance between the sensitized groups and its corresponding control.

To further examine the impact of OVA challenges on various cell types, including eosinophils, interstitial macrophages (IM), alveolar macrophages (AM), neutrophils, total conventional dendritic cells (DCs), and CD11b^+^/CD103^−^ and CD11b^−^/CD103^+^ DC subsets, cells were isolated from different lung compartments. Specifically, samples were collected from the small airways (lung parenchyma), bronchoalveolar lavage fluid (BALF), large airways (trachea), and lung-draining lymph nodes (LDLN) 24 h after the final challenge (Figure 1A). Flow cytometry data were obtained from single-cell suspensions, with each data point representing pooled samples from two mice. The gating strategy is shown in Supplementary Figure S1. The frequency of eosinophils was elevated in both allergic groups (OVA-sensitized) challenged two or three times with OVA aerosol compared to control groups (saline-sensitized) exposed to the same challenges (Figure 3A). This increase was observed across all compartments, including the trachea, but not in the LDLN (Supplementary Figure S2A). Further, there was no significant change of neutrophils in both allergic groups compared to their control groups within all different compartments, except for the sensitized group challenged three times with OVA aerosol in BALF (Figure 3B, Supplementary Figure S2B). The population of interstitial macrophages (IM) was significantly increased in both sensitized groups (OVA-challenged two- or three-times) compared to the negative control group in both the lung parenchyma and BALF, with a similar but non-significant trend observed in the trachea (Figure 3C, Supplementary Figure S2C). In contrast, alveolar macrophages (AM) isolated from BALF were significantly reduced in both sensitized groups compared to their respective controls (Figure 3D). No significant changes in alveolar macrophage (AM) frequencies were observed in the lung parenchyma, trachea, or LDLN (Figure 3D, Supplementary Figure S2D). In the lung parenchyma, the total number of conventional DC was significantly increased in the sensitized group challenged twice with OVA aerosol compared to the control. Although not statistically significant, the sensitized group challenged three times showed a numerically higher frequency of total DCs in the lung parenchyma compared to its control. In BALF, a significant increase in total DCs was detected only in the sensitized group that underwent three challenges, compared to its respective control (Figure 3E). In both allergic groups, the frequency of conventional DCs in the trachea and LDLN was lower than in their respective controls, although these differences were not statistically significant (Supplementary Figure S2E). We further analyzed two distinct DC subsets: CD11b^+^/CD103^−^ (CD11b^+^) and CD11b^−^/CD103^+^ (CD103^+^) across different lung compartments. In the lung parenchyma, the CD11b^+^ subset was significantly increased in the allergic group challenged two times compared to its control, whereas no significant change was observed in the sensitized group challenged three times. In contrast, the CD103^+^ subset showed a significant decrease in total DC frequency in both allergic groups compared to their respective controls. In BALF, the frequency of CD11b^+^ DCs was lower in both allergic groups compared to controls, although this difference was not statistically significant. In contrast, the CD103^+^ subset was significantly reduced within the total DC population in both allergic groups. No significant changes were observed in either subset within the trachea or LDLN in both allergic groups relative to their respective controls (Figure 3F and Supplementary Figure 2F).

### The number of challenges affects the cytokine profile in EAIAD

**Figure 4:**
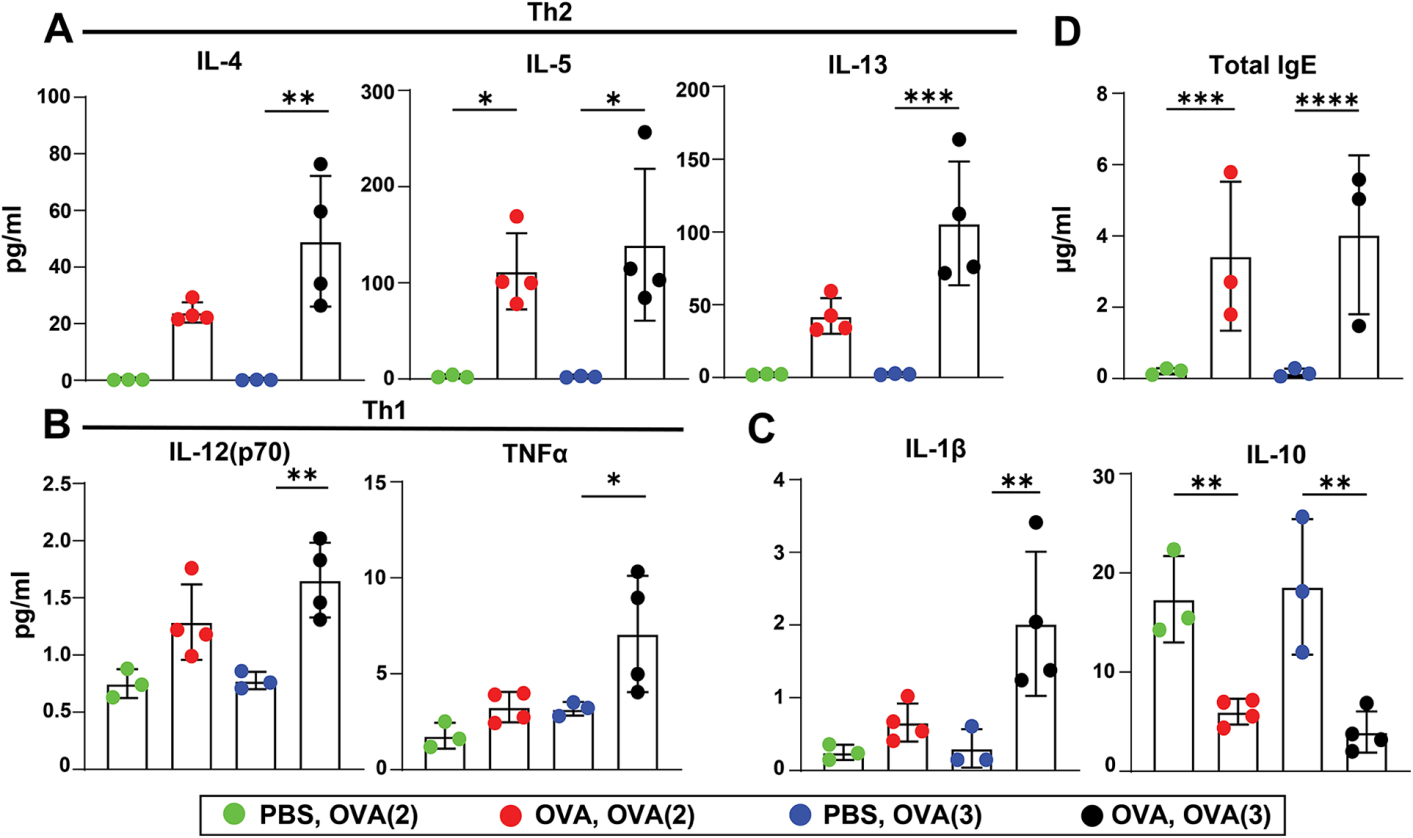
Cytokine and IgE levels in mice with EAIAD. Mice were treated as described in Figure 1A. **(A)** Th2-associated cytokines in bronchoalveolar lavage fluid (BALF): IL-4, IL-5, IL-13; **(B)** Th1-associated cytokines in BALF: IL-12p70, TNFα; **(C)** IL-1β and IL-10; **(D)** total IgE in serum. Data are shown as mean ± SEM. For panels A–C, *n* = 3 for control groups and *n* = 4 for sensitized groups. For panel D, *n* = 3 for all groups. *p < 0.05, **p < 0.01, ***p < 0.001 and ****p < 0.0001.

The concentrations of Th2-associated cytokines in BALF, including interleukin-4 (IL-4), IL-5, and IL-13, were elevated in both sensitized groups challenged two or three times with OVA aerosol compared to their respective controls (Figure 4A). Notably, the Th2 response was more pronounced in the three times challenged model, particularly for IL-4 and IL-13. Th1-associated cytokines, such as IL-12(p70) and TNFα, were significantly increased in the sensitized group challenged three times with OVA aerosol compared to controls (Figure 4B). Consistent with a better equalized response in the three-times challenged model, IL-1β levels were significantly elevated in this group, whereas the anti-inflammatory/regulatory cytokine IL-10 was significantly reduced in both allergic groups compared to controls (Figure 4C). Additionally, serum levels of total IgE were significantly increased in both sensitized groups challenged twice or three times with OVA aerosol compared to their respective controls (Figure 4D).

### Bio-mimetic nanocarriers and their effects on lung function in induced EAIAD

Treatment groups received either virosomes (Viro) or liposomes (Lipo) alone, or virosomes (Viro-OVA) and liposomes (Lipo-OVA) conjugated with OVA, administered subcutaneously on days 42 and 49. Three weeks later, two OVA aerosol challenges were performed on days 70 and 71 (Figure 2A).

After thoroughly characterizing and optimizing our allergy model, we investigated the potential immunomodulatory effects of bio-mimetic nanocarriers, such as liposomes and virosomes, with OVA and TLR7/8 agonist 3M-052 anchored to their surface. TEM imaging of different nano carrier types revealed circularbio-mimetic nanoparticles, often with diameters around 100 nm and smaller (Figures 2B,C). Although cellular immunity was more pronounced in the three times challenged group, we opted for the two times challenged model to ensure consistency in lung function measurements. The additional challenge increased measurement difficulties and the risk of animal loss, making the two times challenged model a more reliable choice.

Animals underwent allergic sensitization as previously described, with a pre-immunization step (27, 28) using inactivated virus on day 0, followed by three subcutaneous OVA injections on days 21, 28, and 35 for the positive control group, while the negative control group received saline injections.

Airway resistance (Rrs) was significantly elevated in the OVA (positive control), Viro, Lipo, and Viro-OVA groups during methacholine challenge compared to the negative control. In contrast, the Lipo-OVA group showed no significant difference from the negative control. However, Rrs was significantly lower in the Lipo-OVA group compared to the positive control, indicating a partial therapeutic effect.

Similarly, all groups except for the LIPO OVA group showed significant elevation in tissue resistance (RN) compared to the negative control. Rn in the Lipo-OVA group was also significantly lower than in the positive control, further supporting improved lung mechanics.

Regarding tissue damping (G), a significant increase was observed in the non-treated positive control group as well as in the Lipo and Viro-OVA-treated groups compared to the negative control. In contrast, the Viro and Lipo-OVA-treated groups did not differ significantly from the negative control. All treatment groups and the negative control differed significantly from the positive control, with the strongest reductions observed in the Lipo-OVA and Viro groups.

FEV0.1 was significantly reduced in the positive control, Viro-OVA, Lipo-OVA, and Viro groups, while the Lipo-OVA group showed no significant change compared to the negative control. All treatment groups showed no significant difference compared to the positive control.

Likewise, PEF was significantly decreased in the positive control, Viro- and Lipo-treated groups compared to the negative control. In contrast, the Viro-OVA and Lipo-OVA groups did not differ significantly from the negative control. However, none of the treatment groups showed a statistically significant difference compared to the positive control (Figure 2D).

**Figure 5:**
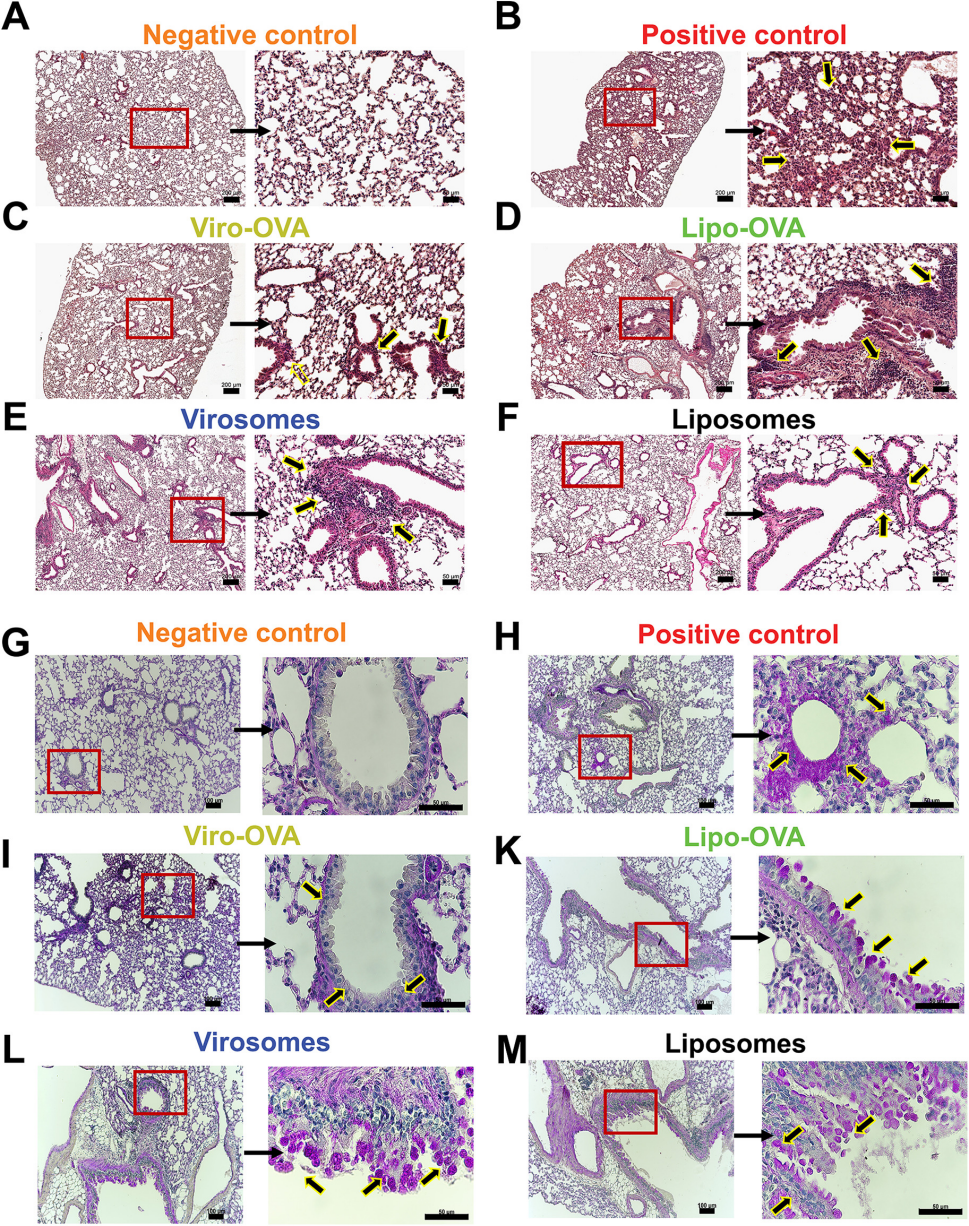
Lung histology in mice with EAIAD following nanoparticle treatment. Hematoxylin and eosin (H&E) staining in paraffin-embedded lung sections from: **(A)** negative control, **(B)** positive control, d**(C)** Viro-OVA-treated, **(D)** Lipo-OVA-treated, **(E)** virosome-treated and **(F)** liposome-treated mice. Left panels: overview images, scale bar: 200 μm. Left panels: overview images 5x magnification (scale bar: 200 µm). Right panels: magnified insets, 20x magnification (scale bar: 50 µm), showing regions of increased inflammatory cell infiltration (arrows). **(G–M**) Periodic acid- Schiff (PAS) staining in paraffin-embedded lung sections from: **(G)** negative control, **(H)** positive control, **(I)** Viro-OVA-treated, **(J)** Lipo-OVA-treated, **(K)** virosome-treated and **(L)** liposome-treated. Left panels: overview images 10x magnification (scale bar: 100μm). Right panels: magnified insets, 60x magnification, (scale ba*r* = 50 µm), showing regions of mucus producing cells (arrows).

To further assess lung structure across the different groups, we performed H&E staining. All groups exhibited noticeable cell infiltration compared to the negative control (Figure 5A). In the positive control group, infiltrating cells were widely distributed throughout large areas of lung tissue (Figure 5B).

In contrast, groups treated with bio-mimetic nanoparticles showed a reduction in cellular infiltrates; however, no major differences were observed between the individual treatment groups (Figures 5C–F). PAS-stained lung sections revealed PAS-positive epithelial cells and luminal material in the positive control group, indicating visible mucus production and goblet cell activity (Figure 5H). In contrast, the negative control group exhibited minimal PAS-positive staining in airway epithelial regions or lumens (Figure 5G). Among the treatment groups, PAS-positive staining was detected in the airways of mice treated with Viro-OVA or Lipo-OVA (Figures 5I,K), as well as in groups that received virosomes or liposomes alone (Figures 5L,M).

### Effect of bio-mimetic nanoparticle treatments on eosinophils, macrophages, and dendritic cells in different lung compartments in EAIAD

**Figure 6:**
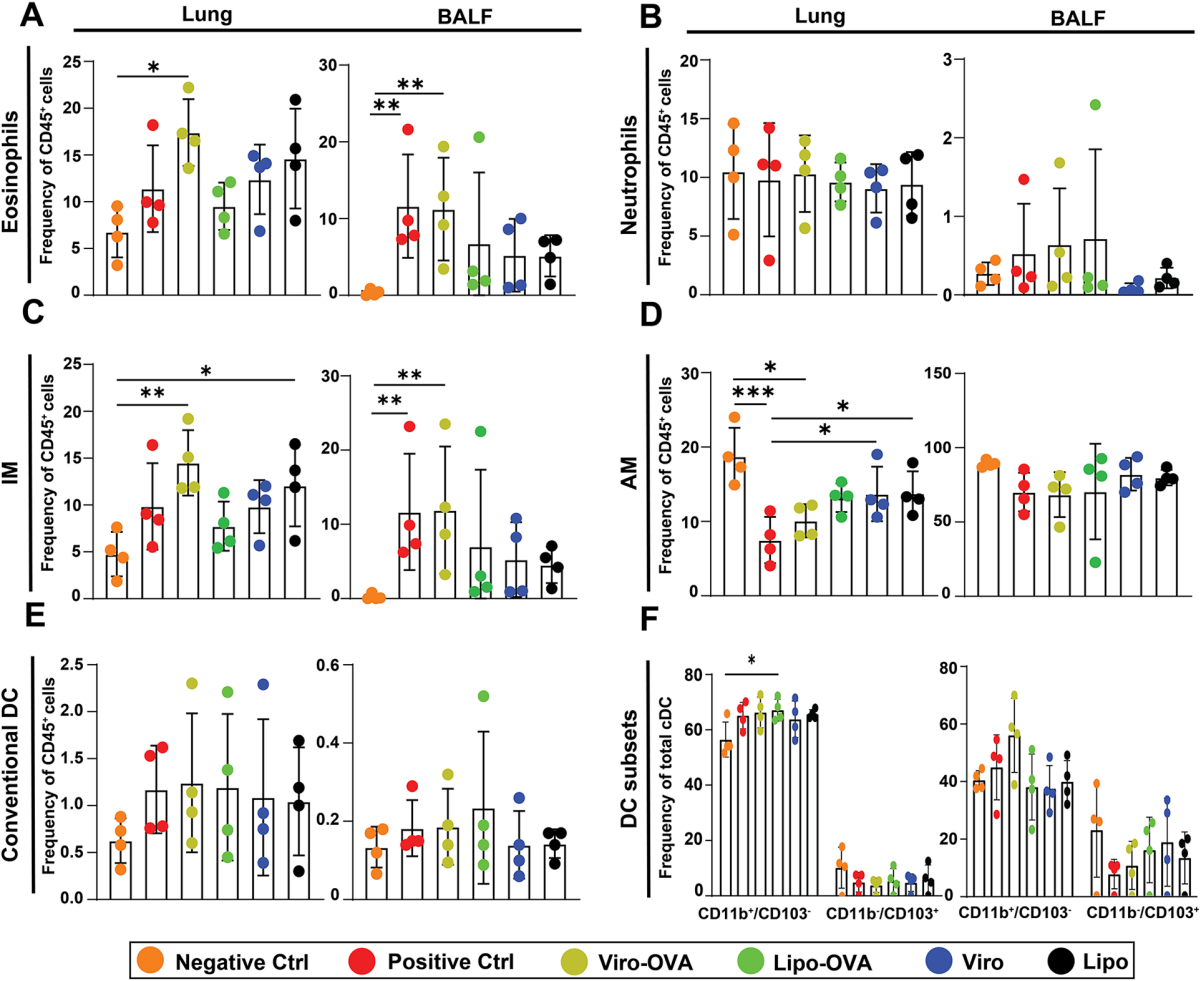
Immune cell profiling in lungs and BALF of nanoparticle-treated mice with EAIAD. Flow cytometric analysis of immune cell subsets (as % of CD45^+^ cells) in lung tissue and bronchoalveolar lavage fluid (BALF) following treatment as described in Figure 4A. **(A)** eosinophils, **(B)** neutrophils, **(C)** interstitial macrophages (IM), **(D)** alveolar macrophages (AM), **(E)** total conventional dendritic cells (cDCs), and **(F)** dendritic cell subsets (CD11b^+^/CD103^−^ and CD11b^−^/CD103^+^). Data shown as mean ± SEM. Each data point represents a pooled sample from two animals, resulting in *n* = 4 pooled samples per group. *p < 0.05, **p < 0.01 and ***p < 0.001.

To assess the cellular immune response following treatment with bio-mimetic nanoparticles, we analyzed leukocyte subsets across lung compartments in the EAIAD model. In the lung parenchyma, eosinophil frequencies were significantly elevated in the Viro-OVA group compared to the negative control. In the BALF, both the positive control and the Viro-OVA group showed significantly increased eosinophils relative to the negative control, confirming ongoing airway inflammation. While the Lipo and Viro groups also showed eosinophil levels closer to baseline, these differences were not significant compared to the positive control. No other groups exhibited statistically significant changes in eosinophil levels in the lung or BALF, trachea or LDLN (Figure 6A and Supplementary Figure S3A).

In terms of neutrophils there was no significant change in frequency within all compartments following any treatment (Figure 6B and Supplementary Figure S3B).

For interstitial macrophages (IM), both the Viro-OVA and Lipo-treated groups displayed significantly higher frequencies compared to the non-treated positive control in the lung, suggesting an enhanced inflammatory profile. In BALF, IM were significantly elevated in the positive control and Viro-OVA groups compared to the negative control. In the trachea there was a significant increase in the Lipo-treated group compared to the negative control, while in LDLN IM frequencies did not differ significantly between the groups (Figure 6C and Supplementary Figure S3C).

Alveolar macrophage (AM) frequencies were significantly reduced in the positive control group and in the Viro-OVA treated group compared to the negative control in the lung. Treatment with Viro and Lipo significantly increased AM frequencies compared to the positive control, indicating a partial restoration of this population. In the trachea and the LDLN AM frequencies did not differ significantly between groups (Figure 6D and Supplementary Figure S3D).

Total conventional dendritic cell (cDC) frequencies did not differ significantly among groups across all lung compartments (Figure 6E and Supplementary Figure S3E). However, analysis of dendritic cell subsets revealed a significant reduction of CD11b^+^CD103^−^ DCs in the Lipo-OVA group compared to the negative control in the lung parenchyma. No significant changes were observed in the other tested lung compartments (Figure 6F and Supplementary Figure S3F).

Overall, the treatments showed compartment- and cell type–specific effects, with no formulation achieving consistent normalization of inflammation-related parameters.

### Cytokine and immunoglobulin profiles reveal treatment-specific effects in allergic mice

**Figure 7:**
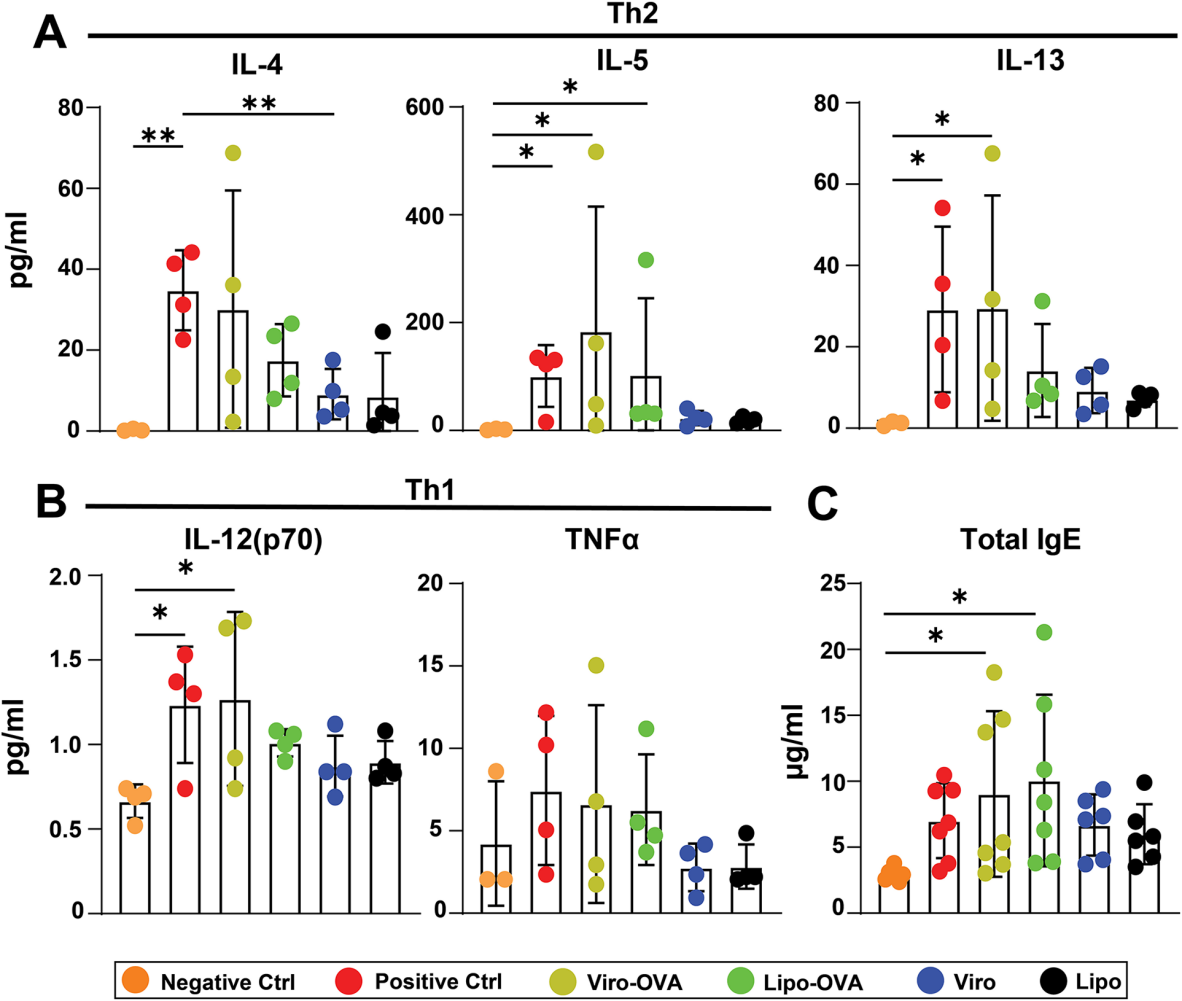
Cytokine and IgE responses following nanoparticle treatment in mice with EAIAD. Mice were treated as described in Figure 2A. **(A)** Th2-associated cytokines in bronchoalveolar lavage fluid (BALF): IL-4, IL-5, IL-13; **(B)** Th1-associated cytokines in BALF: IL-12p70, TNFα; **(C)** IL-1β and IL-10; **(D)** total IgE in serum. Data shown as mean ± SEM; For panels A–B: *n* = 4 mice per group. For panel C: *n* = 7–10 mice per group. *p < 0.05, **p < 0.01, ***p < 0.001.

To assess immunomodulatory effects of the nanoparticle formulations, we measured key Th2- and Th1-associated cytokines and immunoglobulins in BAL fluid and serum. As expected, in BALF IL-4, IL-5, and IL-13 levels were significantly elevated in the non-treated positive control compared to the negative control, confirming a Th2-skewed immune response. Treatment with Viro-OVA significantly increased IL-5 and IL-13 relative to the negative control, whereas Lipo and Viro treatments did not significantly alter IL-5 levels. IL-4 levels remained lower in all treatment groups compared to the positive control, though this reduction was not statistically significant, except for Viro only. No treatment group showed a significant reduction in Th2 cytokines compared to the positive control (Figure 7A).

For Th1-associated cytokines, IL-12(p70) was significantly elevated in both the positive control and the Viro-OVA group compared to the negative control. No treatment group exhibited a significant difference in IL-12(p70) compared to the positive control. TNF*α* levels showed no statistically significant differences across all groups (Figure 7B).

In serum, total IgE levels were elevated in the Viro-OVA and Lipo-OVA treatment groups compared to the negative control. However, no statistically significant differences were observed between treatment groups and the positive control group (Figure 7C).

To further characterize the systemic humoral immune response, we also measured total serum levels of IgA, IgM, IgE, IgG1, IgG2a, and IgG2b (Supplementary Figure S4). Virosome treatment (Viro) significantly increased IgA and IgM levels compared to both control groups. Additionally, both virosome- and liposome-based formulations coupled to OVA (Viro-OVA and Lipo-OVA) induced elevated IgG1 concentrations compared to controls, with Viro-OVA reaching significance vs. all groups except Lipo-OVA. IgG2a levels were significantly elevated in both Viro-OVA and Lipo-OVA groups compared to controls, and to a lesser extent in the Lipo group alone. A similar trend was observed for IgG2b, where Viro-OVA showed significantly higher levels than both control groups. These data indicate that while IgE changes were limited to specific groups, virosome- and liposome-based treatments triggered broader isotype switching and humoral activation beyond IgE, particularly affecting IgG subclasses and IgM.

In summary, although no treatment resulted in a statistically significant reduction in Th2 cytokines or IgE compared to the allergic control, the virosome- and liposome-based formulations, particularly when coupled to OVA, induced broader humoral immune responses characterized by isotype switching to IgG1, IgG2a, and IgG2b. Notably, virosomes alone stimulated elevated levels of IgE, IgA, and IgM, highlighting their intrinsic immunostimulatory potential. In contrast, liposomes alone induced a more limited response, primarily affecting IgG2a levels. These findings suggest that while individual nanoparticles differ in their innate immunogenicity, both platforms are capable of promoting adaptive immune responses when antigen is present, with virosome-coupled formulations demonstrating the most comprehensive humoral activation.

## Discussion

We have employed an adjuvant-free sensitization model, omitting aluminum hydroxide to better characterize the immune response to a defined experimental allergen. The use of alum in murine asthma studies is a matter of debate due to the chemical's non-physiological nature. It has been demonstrated that alum can induce its own particular inflammatory responses, such as mast cell-independent allergic inflammation. This limits the relevance of the allergic model (31). This design allowed us to evaluate the immunomodulatory capacity of bio-mimetic nanoparticles under conditions of well-characterized immune priming. In the therapeutic protocol, we included a pre-immunization step with inactivated influenza virus to reflect common anti-influenza immunity in humans. This pre-existing immunity may enhance interaction with virosomal carriers but was not intended to serve as an adjuvant during sensitization.

Conventional vaccines and immunomodulators often fail to induce durable immunity due to limited immunogenicity, requiring adjuvants to boost efficacy (32, 33). Aluminum-based adjuvants promote Th2 responses but lack the capacity to induce strong cell-mediated immunity (34, 35). This has led to growing interest in nanoparticle-based subunit vaccines, which mimic viral structures for enhanced safety and immunogenicity (36). Among next-generation adjuvants, TLR agonists, particularly TLR7/8, are promising in allergy immunotherapy due to their ability to promote both humoral and Th1 responses (19, 37, 38).

In this study, we optimized an EAIAD mouse model that mimics allergen-driven asthma without traditional adjuvants, to test bio-mimetic nanocarriers co-delivering the OVA antigen and the TLR7/8 agonist 3M-052 on the same nanoparticle. Comparing OVA aerosol challenges twice and three times, we observed that repeated exposure exacerbated symptoms, with three times challenged mice showing significantly reduced FEV0.1 and PEF, complicating lung function assessments. Therefore, therapeutic efficacy was evaluated in two-times challenged animals to minimize variability and distress.

Histological and flow cytometry analyses confirmed allergic inflammation, particularly eosinophilic infiltration in the lung parenchyma and BALF after OVA challenge. Th2 cytokines (IL-4, IL-5, IL-13) and IgE were elevated, while IL-10 was reduced. Notably, TNF*α* remained elevated 24 h post-challenge, unlike in previous models, possibly due to increased CD11b^+^ DCs, known producers of TNFα (39, 40). We also detected a slight increase in neutrophil frequency in BALF following the three-time OVA challenge, however, this increase remained below 1% of CD45^+^ cells. Given the low relative abundance and lack of absolute cell counts, we refrain from drawing conclusions about neutrophil-driven inflammation in this model. Nevertheless, the data suggests that increasing the number of OVA aerosol exposures may amplify airway inflammation, contributing to greater variability in immune responses. Thus, modulating the challenge regimen offers flexibility for tailoring model severity in future immunotherapy evaluations. We further investigated the therapeutic effects of engineered liposomes and virosomes, both derived from safe, empty influenza envelopes (28, 41, 42). Nanocarriers were loaded with surface-bound OVA and TLR7/8 agonists, and mice were pre-immunized with inactivated influenza virus to simulate prior exposure. Pre-immunization slightly increased CD11b^+^ DCs, eosinophils and total IgE in negative controls. Influenza vaccination has been linked to CD11b^+^ DC expansion, enhancing CD4^+^ T cell priming and antibody responses (43). The presence of CD11b^+^ and CD103^+^ DCs has also been associated with the formation of tertiary lymphoid structures (TLS) in the lung tissue of mice following influenza infection, which play an important role in maintaining long-term immune responses (44). Our results showed a significant reduction in airway resistance in animals treated with OVA liposomes, with the strongest effect when combined with TLR7/8 agonists, excluding viral envelope proteins. Surprisingly, virosomes lacking antigen (Viro) were the second most effective at reducing airway resistance. This improvement in lung function was accompanied by a significant reduction in IL-4 levels in BALF compared to the positive control group. No statistically significant differences were observed in IL-5, IL-13, IL-1β, or TNF*α* levels across treatment groups when compared to the positive control. These data suggest that certain formulations, partially modulate cytokine profiles in a manner consistent with dampened Th2 activity and enhanced Th1 responses, although changes beyond IL-4 did not reach statistical significance. Total IgE was significantly increased in Lipo-OVA and Viro-OVA groups compared to controls, but not in treatments with virosomes or liposomes without OVA. TLR7/8 agonists 3M-052, with either antigen (Lipo-OVA) or viral envelope proteins (Viro), showed a compensatory effect in resolving airway hyperresponsiveness. However, combinations of agonists with antigen and viral envelope proteins (Viro-OVA) or agonists alone (Lipo) showed a less pronounced effect.

These results emphasize the importance of surface composition in nanoparticle design. TLR7/8 agonists 3M-052 alone were effective in promoting Th1 skewing and dampening allergic inflammation, but the immune-modulatory balance was disrupted when combined with both antigen and viral components. Careful formulation is essential to avoid excessive activation and ensure allergen-specific modulation. TLR stimulation without antigen can activate APCs and skew immunity toward Th1, though it may impair cross-presentation (45–47). Numerous studies support the anti-allergic potential of TLR7/8 agonists in airway inflammation models (48–51). Acting through APCs and CD4^+^ T cells, they help counteract Th2-driven allergic responses (52). The TLR7/8 agonist used here, 3M-052, has demonstrated long-lasting immunity in non-human primates (53), and recent studies show it can drive robust dendritic and B cell activation, as well as CD8^+^ T cell responses in mice (54). Still, the innate mechanisms by which 3M-052 orchestrates immune responses remain to be fully elucidated.

While our approach demonstrated measurable immune modulation, the therapeutic interventions did not fully reverse the allergic response in the EAIAD model. Among the candidates, Lipo-OVA consistently improved lung function, significantly reducing airway resistance and preserving forced expiratory volume in 0.1 s (FEV0.1), a sensitive indicator of small airway responsiveness. However, these effects were not mirrored across all immunological endpoints. For instance, total IgE levels remained elevated in Lipo-OVA and Viro-OVA groups, and Th2 cytokine reductions were limited, suggesting incomplete immune reprogramming (Table 1).

**Table 1:** Summary of bio-mimetic nanoparticle effects in the EAIAD model. This table provides a summary of immune responses and lung function parameters that were significantly altered by treatment with bio-mimetic nanoparticles (Viro, Lipo, Viro-OVA, and Lipo-OVA) in the EAIAD mouse model, compared to the non-treated positive control. Directionality of effects is indicated by arrows (↑/↓), and parentheses provide additional clarifications where relevant. Parameters that did not show statistically significant differences, including dendritic cell populations (CD103^+^ and CD11b^+^ DCs), Th2 cytokines (IL-4, IL-5, IL-13), Th1 cytokines (IL-12p70, IL-1β, TNFα), and total IgE, are not displayed in the table.

Parameter	Viro	Lipo	Viro-OVA	Lipo-OVA
Lung Function Improvement	↓ Rrs, Rn, G	↓ Rrs, G	↓ Rrs, G	↓ Rrs, Rn, G
Reduction in Eosinophilic Infiltration	ns	ns	↑ (lung)	ns
Macrophage Populations (IM, AM)	↑ AM (lung)	↑ AM (lung)	ns	ns
Dendritic Cell Populations (CD103+, CD11b+ DCs)	ns	ns	ns	ns
Reduction in Th2 Cytokines (IL-4, IL-5, IL-13)	ns	ns	ns	ns
Increase in Th1 Cytokines (IL-12p70, TNFα, IL-1β)	ns	ns	ns	ns
OVA-reactive IgE Increase	ns	ns	ns	ns
Total IgE Levels	↑	ns	ns	ns
Lung Function Improvement	↓ Rrs, Rn, G	↓ Rrs, G	↓ Rrs, G	↓ Rrs, Rn, G
Reduction in Eosinophilic Infiltration	ns	ns	↑ (lung)	ns
Macrophage Populations (IM, AM)	↑ AM (lung)	↑ AM (lung)	ns	ns
Total IgE Levels	↑	ns	ns	ns

Interestingly, virosomes without antigen also conferred some benefit, underscoring the role of nanoparticle surface composition and immunological context in shaping therapeutic outcomes. Virosomes induced strong systemic responses, including elevated IgA, and IgM, indicating potent innate immunostimulatory properties, but their strong IgE-inducing potential may require caution. In contrast, liposomes alone triggered modest increases in IgG2a, reflecting a potential Th1 bias but limited overall activity. When coupled with antigen, both platforms effectively induced adaptive humoral immunity, with Viro-OVA eliciting the broadest isotype profile, including IgG1, IgG2a, and IgG2b, suggesting it is the most immunologically active formulation. These findings support the view that antigen coupling is critical for directing targeted immunity, while the nanoparticle scaffold contributes distinct baseline immune signals.

These findings align with our observation that, although no treatment led to a statistically significant reduction in Th2 cytokines or total IgE, the virosome- and liposome-based formulations, especially when coupled to OVA, elicited broader humoral immune responses. This included isotype switching to IgG1, IgG2a, and IgG2b. Notably, virosomes alone elevated levels of IgE, IgA, and IgM, suggesting strong intrinsic immunostimulatory potential. In contrast, liposomes alone mainly increased IgG2a, reflecting a more limited but Th1-skewed response. Thus, while the two nanoparticle types differ in their baseline immunogenicity, both platforms are capable of promoting adaptive immune responses when antigen is present, with virosome-coupled formulations demonstrating the most extensive humoral activation.

It is important to interpret the total IgE results with caution. total IgE was measured using a capture ELISA employing anti-IgE monoclonal antibodies. This indirect method may lack the specificity of assays using directly OVA-coated plates. Future studies should implement such antigen-specific assays and incorporate IgG isotyping (e.g., IgG1, IgG2a) to better resolve Th1/Th2 polarization at the humoral level.

Optimizing therapeutic impact may require adjustments in dosing intervals (e.g., extending to 14 days), increasing the number of administrations, or refining antigen-to-adjuvant ratios. The complex and extended timeline of the EAIAD model, including pre-immunization, sensitization, treatment, and challenge phases, introduces variables that can influence immune outcomes and may have contributed to an increased likelihood of non-responders. The pre-immunization step using inactivated influenza virus, intended to simulate pre-existing human immunity, likely contributed to increased variability and may have enhanced baseline immune activation. This was reflected in slightly elevated CD11b^+^ dendritic cells, eosinophils, and total IgE levels even in negative control animals.

Notably, while both the model validation and treatment studies used the same two-dose aerosol challenge protocol, FEV0.1 was more markedly reduced in the treatment study, whereas lung eosinophilia did not differ significantly between allergic and non-allergic mice. These discrepancies may be partially attributed to the immunological effects of pre-immunization or procedural refinements to lung function assessment. Indeed, prior studies have shown that pre-existing influenza exposure can exacerbate airway hyperresponsiveness and promote allergen-specific IgE production following allergen challenge (55, 56). These observations underscore how minor experimental differences can affect disease readouts, but do not undermine the validity or translational relevance of the model.

Furthermore, while we assessed total IgE levels consistently across both studies using an ELISA-based method, we acknowledge that measuring OVA-specific IgE would have provided a more precise and antigen-relevant readout of allergic sensitization. Inclusion of such a measurement in future studies would enhance the mechanistic resolution of our model and better align with established protocols for evaluating allergen-specific immune responses.

A further limitation of our study is the absence of absolute cell counts. Due to pooling of tissue from two animals per sample, use of lungs for multiple downstream applications (e.g., histology, parallel FACS panels), and acquisition based on fixed event thresholds, it was not feasible to extrapolate total organ cellularity. As a result, we report frequencies of immune subsets within the CD45^+^ population. While this approach reliably captures shifts in immune composition, future studies incorporating absolute quantification would provide a more complete picture of inflammatory burden and cell recruitment dynamics in allergic airway inflammation.

Despite these challenges, our findings support the potential of TLR7/8 agonist 3M-052 as an immunomodulatory component for asthma therapy, with the EAIAD model providing a valuable platform to assess disease severity and immune polarization. It is also important to consider the role of long-lived plasma cells in sustaining IgE. Prior studies have shown that chronic allergen exposure can lead to the accumulation of IgE-secreting plasma cells in the bone marrow, which can maintain serological memory independently of ongoing allergen exposure (57). Additionally, work by Eckl-Dorna et al. (58) demonstrated that such long-lived IgE-producing cells can persist even during allergen-specific immunotherapy (AIT), potentially limiting its long-term efficacy (58). While these studies did not utilize nanoparticle-based interventions, recent research has begun to explore this intersection. For example, a study by Hughes et al. (59) applied biodegradable nanoparticles for allergen delivery in a murine model of peanut allergy and reported reduced anaphylactic responses and IgE-associated outcomes, demonstrating the potential of bioengineered AIT to modulate IgE-mediated immunity (59). These findings suggest that combining nanoparticle-based delivery with strategies targeting long-lived IgE plasma cells could further improve therapeutic durability in allergic diseases.

In summary, bio-mimetic nanoparticles incorporating TLR agonists and allergens represent a promising avenue for allergen-specific immunotherapy (AIT). While the tested liposomal and virosomal formulations induced partial immunological shifts and functional improvements, their therapeutic efficacy remains incomplete. These findings provide a foundation for further refinement of nanoparticle-based immunotherapy strategies tailored to chronic allergic airway disease.

## Data Availability

The original contributions presented in the study are included in the article/Supplementary Material, further inquiries can be directed to the corresponding author.
