# Supplementary material for: Modulation of allergic airways disease employing bio-mimetic nanoparticles with TLR agonists
Source: Front Allergy. 2025 Aug 29;6:1633293. doi: 10.3389/falgy.2025.1633293 (PMC12426108; doi:10.3389/falgy.2025.1633293)
Supplement: Supplementary file 1 [file Datasheet1.pdf]

Supplemental figures

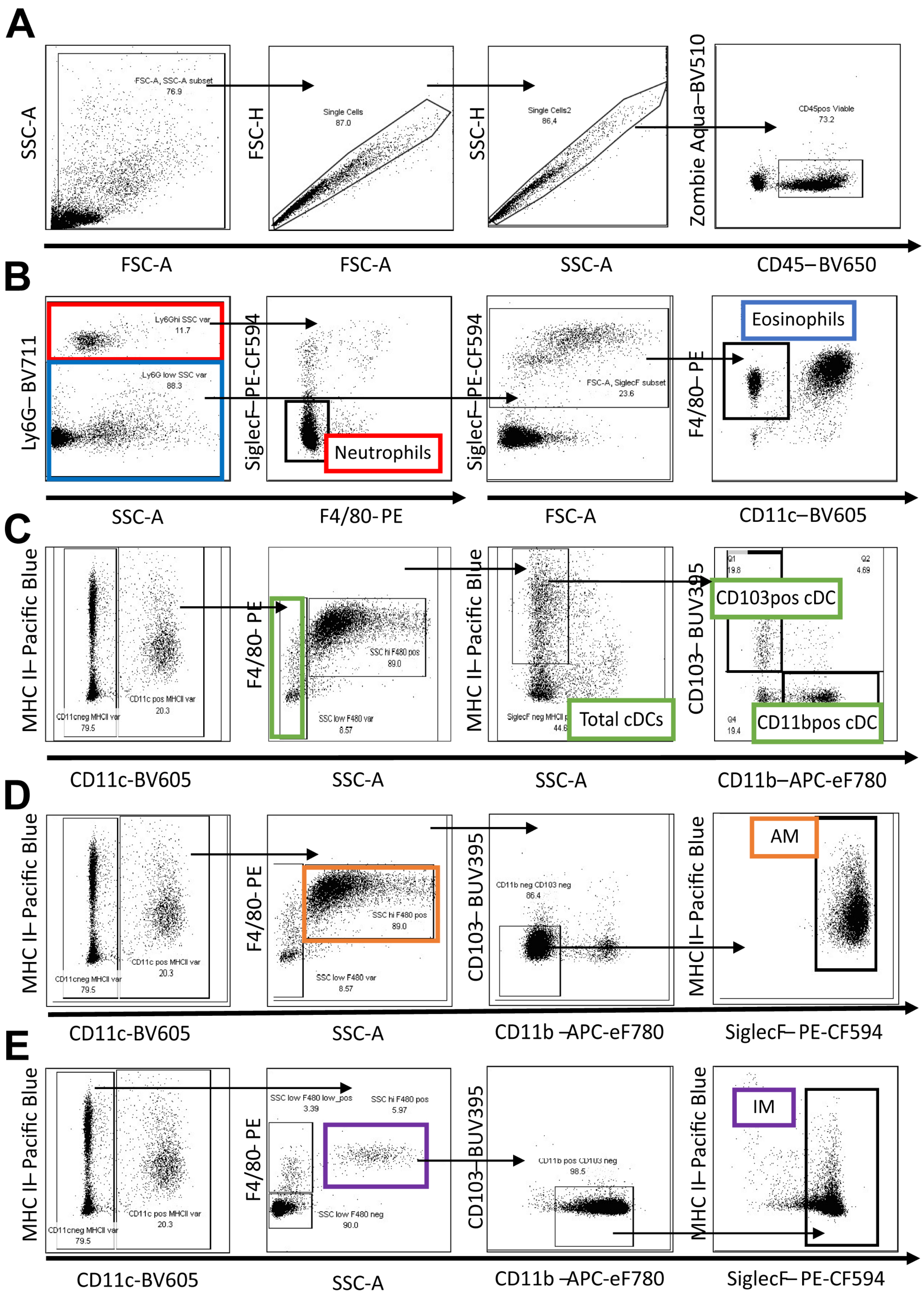

A
SSC-A
FSC-A
FSC-A, SSC-A subset
76.9
FSC-H
Single Cells
87.0
SSC-H
Single Cells2
86.4
SSC-A
Zombie Aqua–BV510
CD45pos Viable
73.2
CD45–BV650
B
Ly6G–BV711
Ly6Ghi SSC var
11.7
Ly6G low SSC var
88.3
SSC-A
SiglecF–PE-CF594
Neutrophils
F4/80-PE
FSC-A, SiglecF subset
23.6
FSC-A
F4/80–PE
Eosinophils
CD11c–BV605
C
MHC II–Pacific Blue
CD11cneg MHCII var
79.5
CD11c pos MHCII var
20.3
CD11c-BV605
F4/80–PE
SSC hi F480 pos
89.0
SSC low F480 var
8.57
SSC-A
SiglecF neg MHCII
Total cDCs
CD103–BUV395
Q1
19.8
Q2
4.69
CD103pos cDC
Q4
19.4
CD11bpos cDC
CD11b–APC-eF780
D
MHC II–Pacific Blue
CD11cneg MHCII var
79.5
CD11c pos MHCII var
20.3
CD11c-BV605
F4/80–PE
SSC hi F480 pos
89.0
SSC low F480 var
8.57
SSC-A
CD103–BUV395
CD11b neg CD103 neg
86.4
CD11b –APC-eF780
MHC II–Pacific Blue
AM
SiglecF– PE-CF594
E
MHC II–Pacific Blue
CD11cneg MHCII var
79.5
CD11c pos MHCII var
20.3
CD11c-BV605
F4/80–PE
SSC low F480 low_pos
3.39
SSC hi F480 pos
5.97
SSC low F480 neg
90.0
SSC-A
CD103–BUV395
CD11b pos CD103 neg
98.5
CD11b –APC-eF780
MHC II–Pacific Blue
IM
SiglecF– PE-CF594

**Figure S1.** Gating strategy for key immune cell populations during EAIAD. (**A**) Initial gating strategy for live, single CD45$^+$ leukocytes. (**B**) Identification of neutrophils and eosinophils. (**C**) Gating of total conventional dendritic cells (cDCs), including CD103$^+$ and CD11b$^+$ cDC subsets. (**D**) Identification of alveolar macrophages (AM). (**E**) Identification of interstitial macrophages (IM). Populations were analyzed in different respiratory compartments including lung parenchyma (Lung), bronchoalveolar lavage fluid (BAL), trachea, and lung-draining lymph nodes (LDLN), based on expression of Ly6G, MHC II, CD11b, CD11c, CD103, F4/80, and SiglecF.

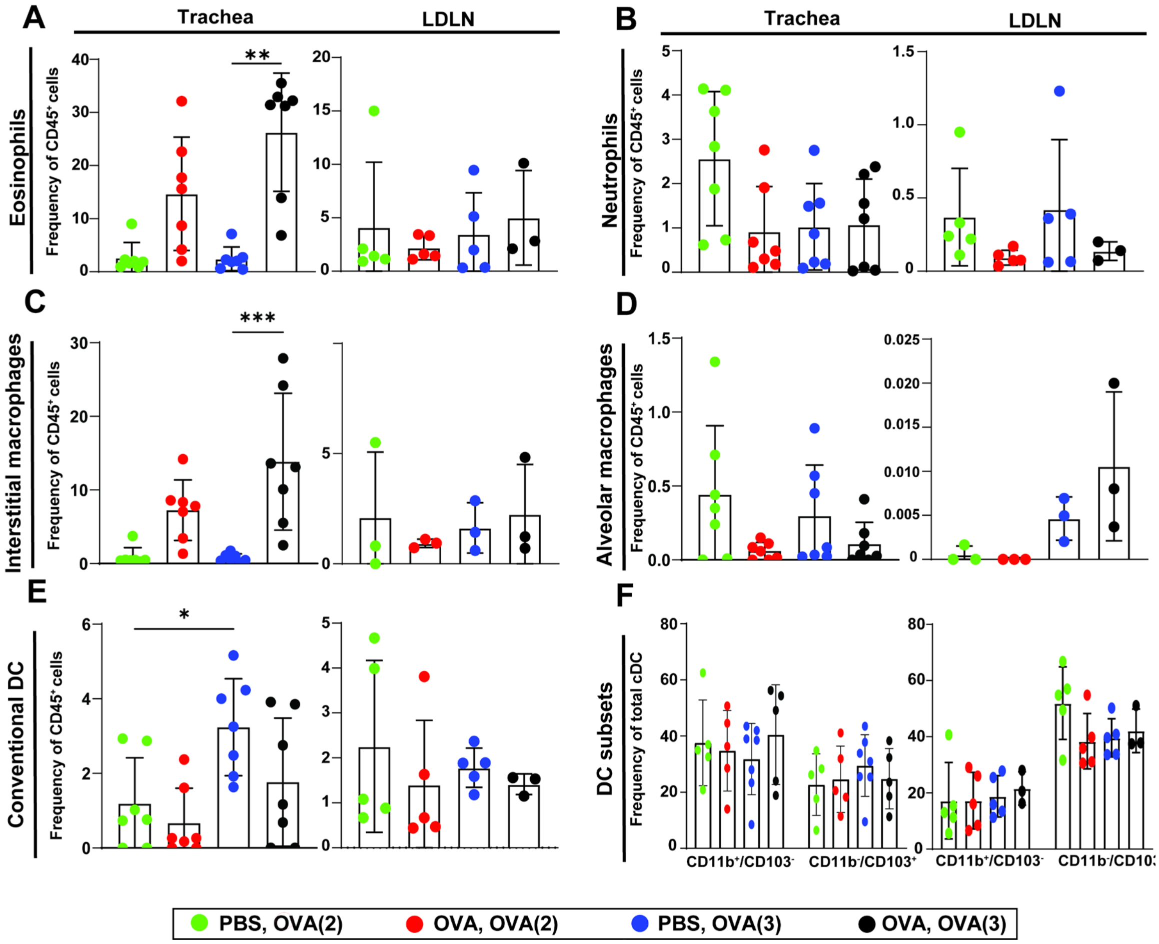


**Figure S2** Immune cell profiles in trachea and LDLN after OVA challenges. Flow cytometric analysis of immune cell frequencies (within CD45$^+$ cells) in sensitized and control mice after two or three OVA aerosol challenges (see Figure 1A). Frequencies of immune cell subsets within total CD45$^+$ cells are shown for: (**A**) eosinophils, (**B**) neutrophils, (**C**) interstitial macrophages (IM), (**D**) alveolar macrophages (AM), (**E**) total conventional dendritic cells (cDCs), and (**F**) dendritic cell subsets (CD11b$^+$/CD103$^-$, and CD11b$^-$/CD103$^+$). Each data point represents a pooled sample from two animals, resulting in n = 7 pooled samples per group, except in panels E and F, where n = 5 pooled samples due to technical limitations. Data shows mean ± SEM. *p<0.05, **p<0.01, ***p<0.001 and ****p<0.0001 show significance between the sensitized groups and its corresponding control.

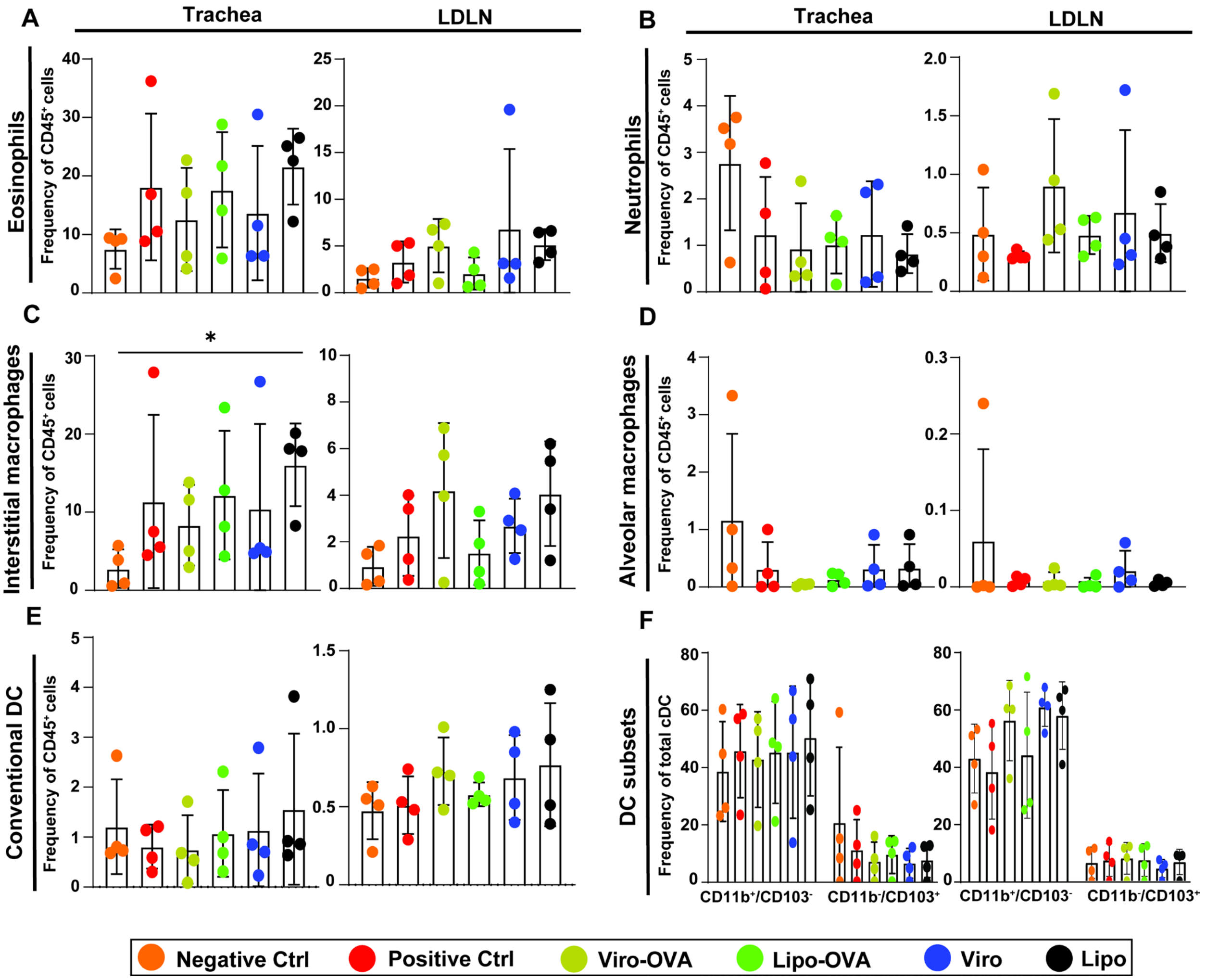


**Figure S3**. Immune cell profiling in trachea and LDLN of nanoparticle-treated allergic mice. Flow cytometric analysis of immune cell subsets (as % of CD45$^+$ cells) following treatment as described in Figure 4A. (**A**) eosinophils, (**B**) neutrophils, (**C**) interstitial macrophages (IM), (**D**) alveolar macrophages (AM), (**E**) total conventional dendritic cells (cDCs), and (**F**) dendritic cell subsets (CD11b$^+$/CD103$^-$ and CD11b-/CD103$^+$). Data shown as mean ± SEM. Each data point represents a pooled sample from two animals, resulting in n = 4 pooled samples per group. *p<0.05, **p<0.01 and ***p<0.001.

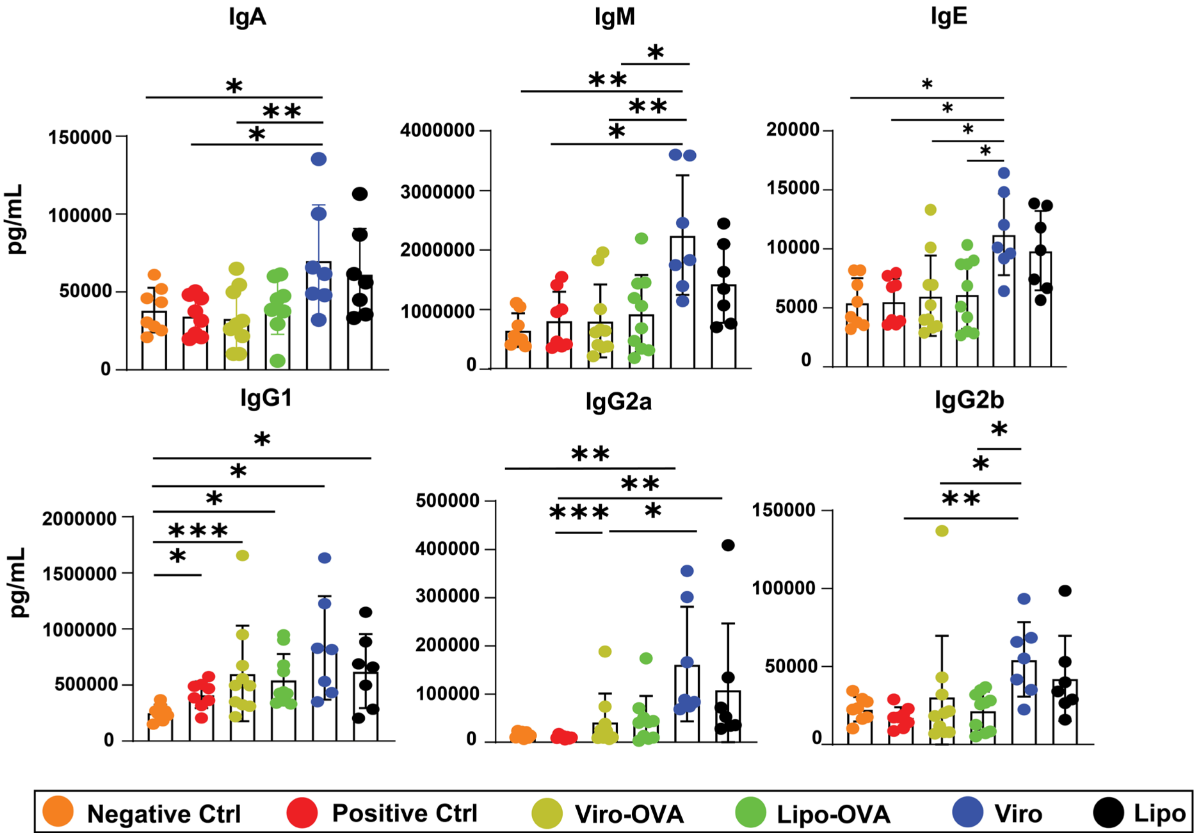


**Figure S4**. Concentration of IgA, IgM, IgE, IgG1, IgG2a and IgG2b in blood serum of un-treated negative and positive controls (Ctrl, OVA) and of groups treated with virosome only (Viro), liposome only (Lipo), virosome and liposome coupled to OVA (Viro-OVA, Lipo-OVA). Data shows mean ± SEM; n=4-7 animals per group. *p<0.05, **p<0.01 and ***p<0.0001.

**Supplementary Table 1**

*Composition and physicochemical characterization of bio-mimetic nanoparticle formulations*

| Formulation | Hemagglutinin (HA) [µg/mL] | OVA-DPPE [µg/mL] | Z-Average [nm] | PDI | IE% (OVA) |
|---|---|---|---|---|---|
| Virosomes | 318.4 | 0.00 | 98.95 | 0.164 | - |
| OVA-Virosomes | 216.8 | 24.43 | 94.96 | 0.094 | 91.8% |
| OVA-Liposomes | 0.00 | 26.74 | 94.63 | 0.081 | 91.1% |
| Liposomes | 0.00 | 0.00 | 109.4 | 0.228 | - |

**Table S1. Composition and physicochemical characterization of biomimetic nanoparticle formulations.** Detailed preparation of virosome- and liposome-based carrier systems, including total formulation volume and the quantities of key components used: Decomposed virus solution, consisting primarily of influenza-derived membrane protein, hemagglutinin (HA), was used as the base for virosomal formulations. OVA-functionalized carriers were generated by covalently anchoring OVA-conjugated 1,2-

dipalmitoyl-sn-glycero-3-phosphoethanolamine (OVA-DPPE) into the lipid bilayer during formulation.  Z-average values and polydispersity index (PDI) were determined by dynamic light scattering (DLS) in PBS at 25°C using the cumulants analysis method. Since OVA was incorporated on the nanoparticle surface and not encapsulated in the lumen, surface integration efficiency (IE%) was calculated by quantifying unbound OVA in the supernatant via dot blot assay, using a standard calibration curve for signal interpolation. HA and OVA-DPPE concentrations are presented as the mean values derived from semi-quantitative analysis based on standard curves.
